# Single Center Retrospective Analysis of Cerebral Aneurysms from a Patient Sample Data Collection at a Comprehensive Stroke Center

**DOI:** 10.51894/001c.34494

**Published:** 2022-09-06

**Authors:** Brian Fiani, Frank DeStefano, Alessandra Cathel, Marisol Soula, Taylor K. Reardon

**Affiliations:** 1 Department of Neurosurgery Weill Cornell Medical Center/New York Presbyterian Hospital, New York, NY; 2 College of Osteopathic Medicine Kansas City University, Kansas City, MO; 3 Department of Neurosurgery Desert Regional Medical Center, Palm Springs, CA; 4 Grossman School of Medicine New York University, New York, NY; 5 Kentucky College of Osteopathic Medicine University of Pikeville, Pikeville, KY

**Keywords:** Cerebral aneurysm, Hunt and Hess, Fisher, Aneurysmal rupture, Spontaneous subarachnoid hemorrhage

## Abstract

**INTRODUCTION:**

Institutional self-monitoring of cerebral aneurysm data should occur regularly. The objective of this retrospective single center study was to examine the reproducibility of a data collection and analytic method to examine cerebral aneurysm characteristics and trends.

**METHODS:**

A single center retrospective analysis was performed from 2018 to 2021 of the most recent 100 patient presentations with a newly diagnosed cerebral aneurysm. Data collection included patient demographics, radiographic features, ruptured or unruptured status, location, grading scale, treatment strategy, survival, and length of stay, which were extracted and presented in tabular form and analyzed for overall trends.

**RESULTS:**

Of the collected 100 patients meeting ICD-10 criteria, 10 (10%) patients were excluded due to having been previously diagnosed at the institution and not meeting the criteria of a new discovery of cerebral aneurysm for inclusion. The remaining 90 sample patients presented with newly diagnosed aneurysms to the authors’ Emergency Department between 2018 and 2021. Most patients were between the ages of 25 and 65 with 55 (61%) patients identifying themselves as female sex. Of the 90 eligible sample patients, 59 (66%) had aneurysms that were not ruptured. Eighty-eight (97.7%) patients had cerebral aneurysms that were < 7mm in size. The most common location for aneurysms was in the anterior cerebral circulation, with identification of 27 middle cerebral artery aneurysms. Length of stay (LOS) ranged from 0-171 days with a mean of 11.97 days (SD = 19.9). Of the seven (7.7%) patients who expired, four (57%) experienced spontaneous subarachnoid hemorrhages, with two (29%) occurring in the anterior communicating artery and one (14%) in the left middle cerebral artery and basilar artery respectively.

**CONCLUSIONS:**

The typical presentation of a cerebral aneurysm is unruptured with a pre-dominance in middle-aged females. Our findings are congruent with the literature regarding the location of the aneurysm originating in the anterior circulation. However, most aneurysms in our clinical cohort were located on the MCA/ICA in contrast to the literature reported (i.e., most anterior communicating artery). Of those patients who presented unruptured, outpatient follow-up and routine monitoring were appropriate with medical management in the setting of small aneurysms. The risk of progression and subsequent rupture was relatively small in this patient cohort. Multi-year examinations of single institution comprehensive stroke centers regarding cerebral aneurysms would enable researchers to conduct regional analyses and comparisons to national and international trends.

## INTRODUCTION

As of 2011, an estimated 6.5 million Americans have developed an unruptured brain aneurysm because of a weakened tunica intima (layer of blood vessel closest to the lumen) that leads to a ballooning of the cerebral arteries.^1^ Most of these aneurysms are asymptomatic yet, in rare cases they can rupture having a tremendous impact on a patient’s health.^1^ Patients with unruptured brain aneurysms present with headaches, drowsiness/confusion, visual deficits, weakness, and facial pain.^1^ If ruptured, the hemorrhage causes the patient to experience severe and sudden headache, nuchal rigidity (neck stiffness), photophobia, loss of consciousness, seizures, cranial nerve palsy (a focal deficit linked to a cranial nerve), and cardiac arrest.^2^ The survival rate post rupture has been about 60% with 66% of those surviving having permanent neurological deficits.^2^ Consequently, research efforts are focused on the diagnosis and treatment of unruptured aneurysms.^2^

Aneurysms are consistently diagnosed with imaging modalities such as computed tomography (CT) and Magnetic Resonance Imaging (MRI), with additional angiographic and blood tests (e.g., lipid profile, thyroid studies, hemoglobin A1c) being implemented when a rupture is suspected. If a rupture is diagnosed the most common treatments include surgical clipping, endovascular coiling, or the use of a flow diverter.^3^ These techniques can also be used preemptively for asymptomatic aneurysms.

However, this practice area remains controversial as the procedural risks of intervening may outweigh any potential benefits.^4^ Institutional self-monitoring should occur regularly to identify the quantity of patients being newly diagnosed with cerebral aneurysm, their collective natural history, and at least simple outcome measures.^4^

### Study Objective

The objective of this retrospective single center study was to examine the reproducibility of a data collection and analytic method to examine cerebral aneurysm characteristics and trends.

## METHODS

After healthcare system IRB approval, the authors conducted single center retrospective analysis of data obtained from a comprehensive stroke center designated facility. The hospital’s patient database was searched using International Classification of Diseases-10 (ICD-10) code I67.1. The goal was to identify the most recent 100 patient presenting with confirmed cerebral aneurysms. Patients with more than one aneurysm were included if one of the aneurysms was identified as part of the recent account visit work up. Patients were excluded if pathologic diagnosis was not confirmed and/or if their aneurysm had been previously diagnosed.

Data collection included patient demographics, radiographic features, ruptured or unruptured status, location, grading scale, treatment strategy, survival, and length of stay, which were extracted and presented in tabular form. Data were summarized for both ruptured and unruptured aneurysms, then combined for analysis. Microsoft Excel was utilized for data sorting and analysis. Categorical data was analyzed for overall trends by third author AC.

## RESULTS

**Figure 1.**
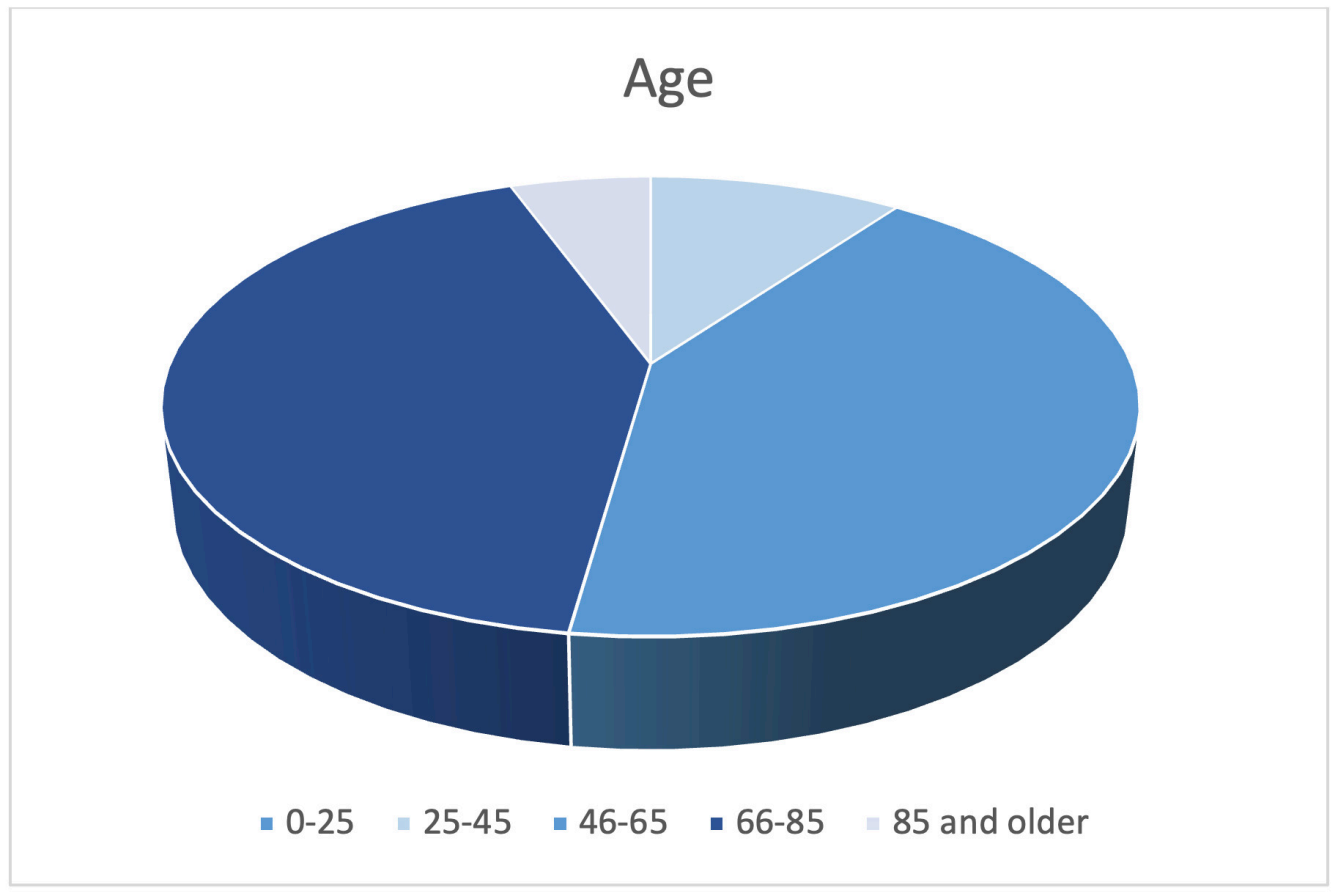
Age distribution of patients with new diagnosis of cerebral aneurysm.

Of the 100 sample patients found to initially meet ICD-10 criteria, 10 patients were excluded due to having been previously diagnosed at our institution and not meeting the criteria of a new discovery of cerebral aneurysm for inclusion. The remaining 90 patients presented with newly diagnosed aneurysms to the Emergency Department at our institution between 2018 and 2021. Two age groups were largely identified making up most presenting patients at 42% equally - those ages 25-45 and those 46-65 (Figure 1).

**Figure 2.**
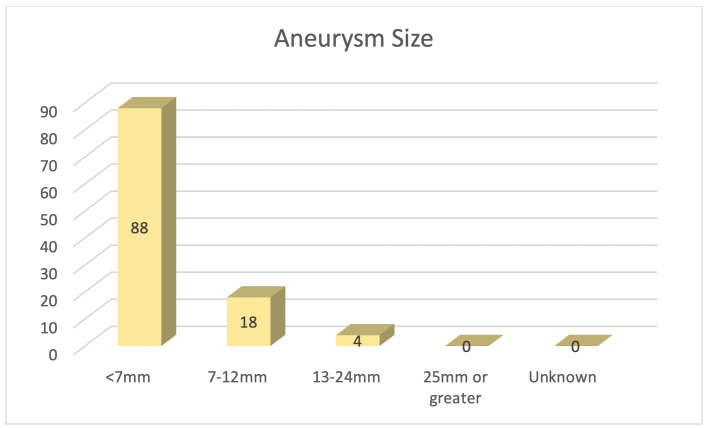
Aneurysm size based on angiographic imaging.

A subset of 55 patients were female, which correlated to roughly 61% of the population identified. Of the 90 patients who presented, 59 (66%) had unruptured aneurysms. Twenty patients presented with more than one aneurysm. 88 of these patients had cerebral aneurysms that were < 7mm in size (Figure 2).

**Figure 3.**
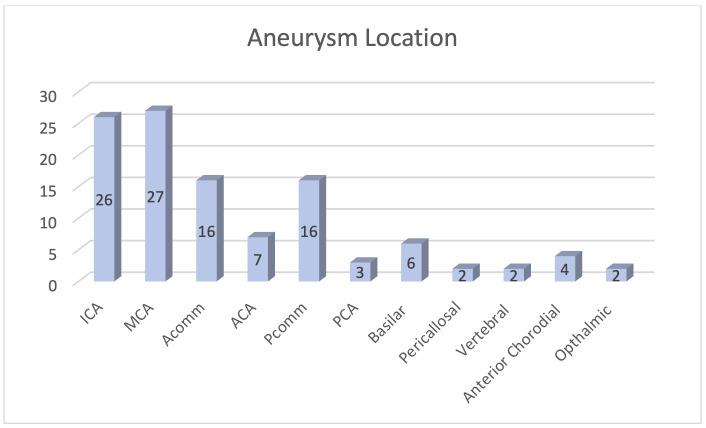
Distribution of aneurysm locations with a majority in the middle cerebral artery (MCA) or the internal carotid artery (ICA).

The most common location for aneurysms was in the anterior circulation, with identification of 27 middle cerebral artery aneurysms, and 16 internal carotid artery aneurysms (Figure 3).

Anterior communicating artery and posterior communicating artery aneurysms occurred at same frequency with 16 noted respectively. Length of stay (LOS) ranged from 0-171 days with a mean of 11.97 days (SD = 19.9).

**Figure 4.**
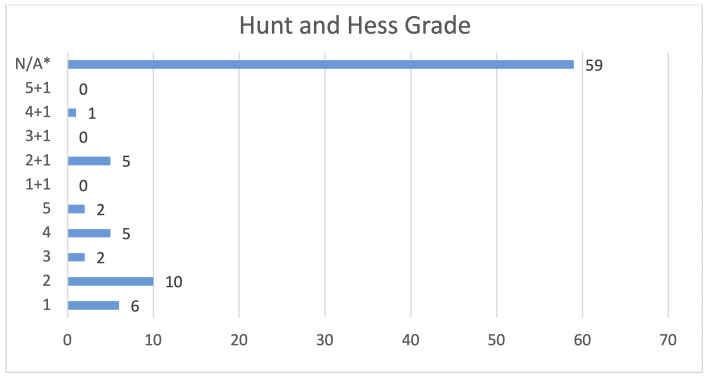
Hunt and Hess Grades recorded for sample patients. *Hunt and Hess Grade not applicable for the 59 patients with unruptured aneurysms.

**Figure 5.**
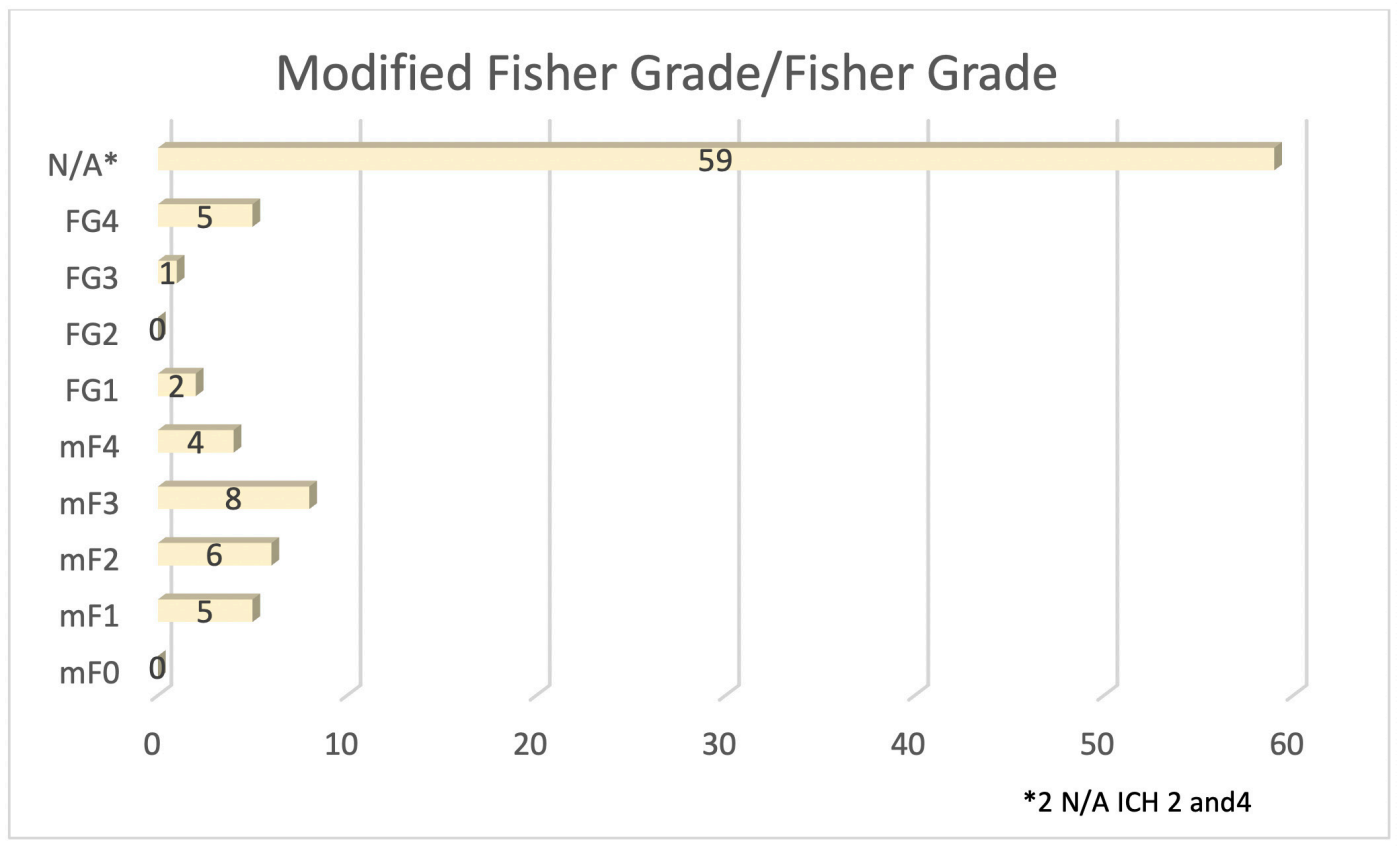
Modified Fisher Grade and Fisher Grade. *These grading scales are not applicable to the 59 patients with unruptured cerebral aneurysms.

Of the seven (7.7%) sample patients who were noted to have expired, four (57%) experienced spontaneous subarachnoid hemorrhages, with two (29%) occurring in the anterior communicating artery and one (14%) in the left middle cerebral artery and basilar artery respectively. These patients experienced an average Hunt and Hess score (longstanding clinical grading system for spontaneous subarachnoid hemorrhage from 1 to 5 with higher scores predictive of poor outcome) of 4.7 and Fisher Grade (classification system for blood on CT head from 1 to 4 predictive of vasospasm) of 4 (**Figures** 4 and 5).

**Figure 6.**
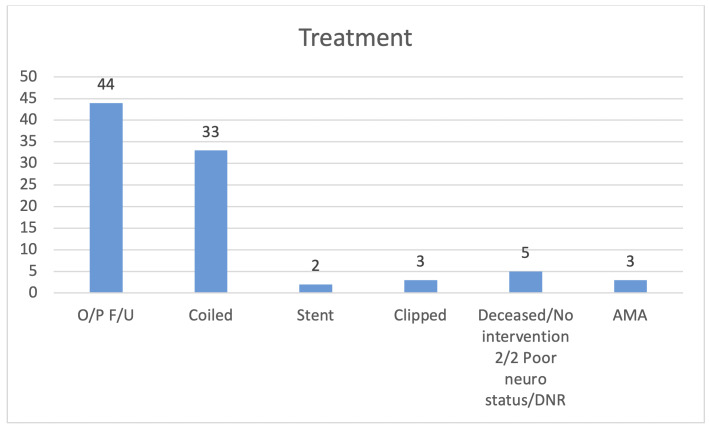
Treatment and management for the patients. (O/P F/U = outpatient follow up, AMA = patient left against medical advice)

Two patients with unruptured aneurysms and one patient with ruptured aneurysm expired. One unruptured aneurysm had a Hunt and Hess Grade 1 and Fisher Grade of 1, with the other not reported due to death from COVID-19 infection. The ruptured aneurysm had both a Hunt and Hess Grade of 4 and Fisher Grade of 4. In total, 33/90 aneurysms were treated with endovascular coil embolism (Figure 6).

A subgroup of 48 patients (53%) were discharged to home with self-care, while 14 (16%) were discharged to home with assistance from a home health organization and 11 (12%) were discharged to a skilled nursing facility.

## DISCUSSION

This study aimed to evaluate the clinical presentation, diagnostic evaluation, and treatment of the most recent 100 newly diagnosed cerebral aneurysms from a single community-based healthcare institution. Fifty-nine (66%) of aneurysm cases presented unruptured. For those unruptured presentations, the aneurysm(s) were discovered incidentally, or the patient came in clinically symptomatic with headache as the most common reported symptom. Subsequent imaging found the aneurysm to be of small size (less than 7 mm) and of internal cerebral artery (ICA) or middle cerebral artery (MCA) origin.

Patients later diagnosed to have a ruptured aneurysm at admission most commonly presented in another study with lethargy, confusion, or mild focal neurological deficits (Hunt & Hess = 3).^5^ Following diagnostic workup, patients presenting with a ruptured aneurysm were found to be of medium size (7-12 mm) with the most common location being of MCA origin. Analysis of consequent subarachnoid hemorrhage most commonly demonstrated a Modified Fisher Grade 4.^6^

The results of our study are congruent with earlier published studies. In both our and another earlier study, the typical presentation of a cerebral aneurysm presents unruptured with a pre-dominance in middle-aged females.^7^ Our findings also agreed with the literature regarding the location of the aneurysm originating in the anterior circulation. However, the majority of aneurysms in our clinical cohort were located on the MCA/ICA in contrast to the literature reported anterior communicating artery.^2^ No specific clinical characteristics or other factors were noted to explain this discrepancy.

Of those patients who presented with unruptured aneurisms, non-hospital follow-up and routine monitoring were appropriate with medical management in the setting of small aneurysms. The risk of progression and subsequent rupture is relatively small in this patient cohort.^8^ In our study, patients presenting with ruptured aneurysms were found to have larger aneurysms than those presenting with unruptured. Our finding matches the International Study of Unruptured Intracranial Aneurysms ISUIA study findings demonstrating a positive association between aneurysm size and future risk of rupture.^9^ Endovascular intervention via coiling was the predominant intervention in our clinical sample for the treatment of ruptured aneurysm as reflected in the current literature.^10^

Current research suggests that the pathophysiology of cerebral aneurysms is likely an additive or multiplicative effect involving several factors.^11–13^ The most studied predictive factors of ruptured aneurysms are size and site. In 1998, the large multicentric retrospective International Study of Unruptured Intracranial Aneurysms (ISUIA) estimated the likelihood of rupture of unruptured intracranial aneurysms and concluded risk of rupture for aneurysms over 10 mm in diameter was approximately 1% per year while smaller than 7 mm was 0% and thus are usually benign.^14^ However, recent publications are challenging this by providing data that small aneurysms (5 mm) can also rupture and other factors such as aneurysm location and patient history are more valuable predictors of rupture.

Aneurysms most commonly form at the weakest points of arteries, the branch points. In these regions, the irregular shape creates an altered hemodynamic environment that causes arterial wall shear stress.^15,16^ 35% of aneurysms form in the anterior communication artery followed by the internal carotid artery, the middle cerebral artery, and lastly, the basilar artery tip.^17,18^ Epidemiological studies have also demonstrated that factors such as alcohol consumption, smoking, hypertension, sex, and family history contribute to the pathogenesis of cerebral aneurysms,^19–22^ in addition to inheritable connective tissue, kidney, endocrine and hemorrhagic disorders.^23–26^

### Study Limitations

The results can not only be used for purposes of risk stratification, but also may aid in highlighting preventive strategies. Due to its retrospective design, another limitation of this study is our exclusion of patients due to missing data. Our findings from a single institution may not be representative of the nation’s population. In summary, this study provides a foundation for future investigation of the neurosurgical management of cerebral aneurysms.

## CONCLUSIONS

Multi-year examination of single institution comprehensive stroke centers regarding cerebral aneurysms could facilitate regional analyses and comparisons to national and international trends. Future considerations include a prospective study investigating the risk-benefit analysis of various endovascular interventions including coiling and flow diversion, or comparison of open surgery versus endovascular therapies. Further studies are needed to strengthen demographic, epidemiologic, and interventional trends at this institution.
